# Cluster of Human Infections with Avian Influenza A (H7N9) Cases: A Temporal and Spatial Analysis

**DOI:** 10.3390/ijerph120100816

**Published:** 2015-01-15

**Authors:** Yi Zhang, Zhixiong Shen, Chunna Ma, Chengsheng Jiang, Cindy Feng, Nivedita Shankar, Peng Yang, Wenjie Sun, Quanyi Wang

**Affiliations:** 1 Beijing Center for Disease Prevention and Control (CDC), Beijing 100013, China; E-Mails: zps347@163.com (Y.Z.); ma_chun_na@126.com (C.M.); yangpengcdc@163.com (P.Y.); 2 Department of Earth and Environmental Sciences, Tulane University, New Orleans, LA 70118, USA; 3 Department of Marine Science, Coastal Carolina University, 301 Allied Drive, Conway, SC 29526, USA; E-Mail: zshen@coastal.edu; 4 Maryland Institute for Applied Environmental Health, School of Public Health in University of Maryland, College Park, MD 20742, USA; E-Mail: cjiang89@umd.edu; 5 School of Public Health & The Western College of Veterinary Medicine, University of Saskatchewan, Saskatoon, SK S7N 5E5, Canada; E-Mail: cindy.feng@usask.ca; 6 Saw Swee Hock School of Public Health, National University of Singapore, Singapore; E-Mail: nivedita_shankar@nuhs.edu.sg; 7 School of Food Science, Guangdong Pharmaceutical University, Zhongshan 528458, China; 8 Department of Global Environmental Health Sciences, School of Public Health and Tropical Medicine, Tulane University, New Orleans, LA 70112, USA

**Keywords:** H7N9, influenza A, GIS, SatScan, space-time, clustering

## Abstract

*Objectives*: This study aims to describe the spatial and temporal characteristics of human infections with H7N9 virus in China using data from February 2013 to March 2014 from the websites of every province’s Population and Family Planning Commission. *Methods*: A human infection with H7N9 virus dataset was summarized by county to analyze its spatial clustering, and by date of illness onset to analyze its space-time clustering using the ESRI^®^ Geographic Information System (GIS) software ArcMap™ 10.1 and SatScan. *Results*: Based on active surveillance data, the distribution map of H7N9 cases shows that compared to the rest of China, the areas from near the Yangtze River delta (YRD) to farther south around the Pearl River delta (PRD) had the highest densities of H7N9 cases. The case data shows a strong space-time clustering in the areas on and near the YRD from 26 March to 18 April 2013 and a weak space-time clustering only in the areas on and near the PRD between 3 and 4 February 2014. However, for the rest of the study period, H7N9 cases were spatial-temporally randomly distributed. *Conclusions*: Our results suggested that the spatial-temporal clustering of H7N9 in China between 2013 and 2014 is fundamentally different.

## 1. Introduction

It is known that influenza A viruses that typically infect and transmit among one animal species sometimes can cross over and cause illness in another species. Novel avian influenza A (H7N9), a newly emerging infectious disease with a high fatality rate in humans [1], has been initially reported in China in March 2013, followed by its wide spread in the eastern provinces of China and the first recorded death in 3 March 2013 [2]. Sequence analyses found that H7N9 viruses were of avian origin, but could adapt to mammalian species [3,4]. This virus normally causes severe respiratory illness in humans, but demonstrates an asymptomatic infection in poultry [5]. Until now, a majority of human cases have been found in China, although sporadic imported cases have been detected in adjacent areas. As the human mortality rate due to H7N9 virus infection is approximately 36% and the virus can occasionally transmit from person to person [6,7], H7N9 poses a potential epidemic threat for China and a pandemic threat for the rest of the world [8].

Describing the epidemiological distribution of cases is the first step in discovering a novel variation in the gene sequence of the influenza virus and acts as the basis for further research. Geographic Information Systems (GIS) are useful tools in mapping the spatial diffusion of infectious diseases, and with related technologies, offer the capacity to conduct space-time analysis, making the epidemiological description more accurate. Thus, it has been widely used in mapping the distribution of infectious diseases and detecting potential case clustering. For example, GIS was used in mapping the Pandemic A (H1N1) in 2009 [9] and the outbreak of Severe Acute Respiratory Syndrome (SARS) [10]. 

A previous study using the human H7N9 case data from March to May 2013 has suggested that the risk of H7N9 varies geographically [3]. However, it is necessary to analyze H7N9 cases data covering all seasons of a whole year, especially for this newly emerging infectious disease for which the spread mechanisms are still unclear, to improve our understanding of the risks of H7N9. Furthermore, disease control polices, such as suspending normal business operations, regular disinfection and the closure of live poultry markets [11,12,13,14], may have affected the spread of the disease and changed its clustering. Therefore, reanalysis using data for the entire period from February 2013 to March 2014 is warranted. 

Here we conducted a GIS analysis of the H7N9 outbreak by focusing on its space-time features, especially its clustering features. This study enhances the knowledge base on the current status of the epidemic and contributes to a greater understanding of the areas where the outbreak was focused. It also helps to raise further hypotheses to explain the current features of the epidemiological distribution of the virus. 

## 2. Materials and Methods

### 2.1. Case Definition

A confirmed H7N9 case is defined as a patient with both an influenza-like illness and with a positive H7N9 laboratory confirmation. Influenza symptoms include fever, cough, headache, muscle aches, generalized malaise or a recent history of exposure to poultry within one week before the onset of symptoms. A positive laboratory confirmation of exposure to H7N9 is made by the “Diagnosis and Treatment Programs of Human Infections with H7N9 virus” issued by the National Health and Family Planning Commission of the People’s Republic of China [15]. The laboratory confirmation is considered positive if either the H7N9 virus was isolated; the H7N9 viral RNA was detected by real-time reverse transcription polymerase chain reaction (rRT-PCR) from a patient’s respiratory specimens or if H7N9 avian flu specific antibody levels increased four times or more in double serum specimens.

### 2.2. Data Source

An epidemiological survey was conducted using a standardized questionnaire for each confirmed case. Demographic information included age, sex, date of onset of symptoms, date of diagnosis and place of residence. This information was collected and released in websites of every province’s population and family planning commission. The study was conducted between 19 February 2013 and 31 March 2014.

### 2.3. Geographic Information System (GIS) Method

The H7N9 virus dataset for cases of human infections is used for this analysis. The dataset is summarized by county in China to analyze spatial clustering, and by date of illness onset at the county level to analyze its space-time clustering. Hot Spot Analysis tool of the ESRI^®^ Geographic Information System (GIS) software ArcMap™ 10.1 was used in this study. This tool allows us to identify where and when the H7N9 virus clusters. It calculates a Getis-OrdGi* statistic [16] for each feature in the GIS analysis according to the properties of its neighbor features. This produces a GizScore and a *p*-value for each feature which is then used to evaluate local clustering of H7N9 cases.

A neighborhood in the analysis is defined by the spatial and temporal distance between features as features are probably correlated to each other according to their spatial and temporal relationships. For example, a H7N9 case probably has a strong correlation with another case if there is a relatively short gap in both space and time between the two cases. This would imply that both cases were most likely caused by the same original source. Where spatial and temporal differences are significant, we may conclude that there is no correlation between these cases. Therefore, it is necessary to define a threshold for spatial and temporal distances where features show the strongest statistically significant correlation. The Spatial Autocorrelation tool and the Incremental Spatial Autocorrelation tool in ArcMap™ 10.1 that analyze the spatial distribution of datasets on a global scale were used for this purpose. 

The threshold spatial distance was defined by analyzing the spatial clustering of H7N9 cases, ignoring all temporal properties, using the Incremental Spatial Autocorrelation tool of ArcMap™. By investigating the statistically significant level of spatial clustering of H7N9 cases at the county level and by defining neighborhoods as ranging from 10 to 590 km at 20 km intervals, a peak significance level of clustering was found that may indicate the threshold distance where spatial clustering is most prominent. A spatial distance of 10 km ensures the closest features to be neighbors while a spatial distance beyond 590 km does not create additional statistically significant clustering.

The analysis was subsequently expanded to a space-time analysis to help define a threshold temporal distance for the clustering of H7N9 cases. The space-time clustering of H7N9 is summarized by date of illness onset at the county level and by generating a threshold distance using the Spatial Autocorrelation analysis tool by varying temporal distance from three to 319 days. A peak of statistical significance of space-time clustering may indicate the threshold temporal distance where clustering is most prominent. In this case, time was defined according to the day the symptoms were observed and not the day the cases were reported.

The Incremental Spatial Autocorrelation and the Spatial Autocorrelation tools calculate a Moran’s I Index value and produce a *z*-score and a *p*-value to test the null hypothesis that the attribute being analyzed is randomly distributed among the features [16]. These tools test whether the dataset is clustered in a statistically significant manner across the whole study area, making them different from the Hot Spot Analysis tool [16]. 

With the threshold spatial and temporal distance defined, a Weights Matrix File was created using the “Generate Spatial Weights Matrix” tool of ArcMap™ to log weights that measure the strength of correlation between features by their spatial and temporal relationship. The generated Weights Matrix File was used to define the parameter “Conceptualization of Spatial Relationships” in the Hot Spot Analysis. 

### 2.4. SatScan Method

The Space-Time scan statistic was also used to explore the spatial-temporal clusters of H7N9 cases. Specifically, a space-time permutation scan statistic model [17] in SatScan [18] (V9.1) was applied. In this model, only case data and location information related to the cases were needed. A cylinder was used to define a potential spatial-temporal cluster, which centered at the county with different spatial radius used to search clusters in spatial and a time period of 1–28 days as the height of the cylinder for temporal cluster. The percentage of cases observed within a cylinder was compared to what would have been expected if the cases were randomly distributed in space and time. The statistical significance of each cluster was calculated based on generalized likelihood ratio (GLR) and Monte Carlo hypothesis testing [17]. In this paper, the simulation was set to 999 times. The *p*-value of 0.05 means that the GLR in this cylinder is higher than 5% of the GLR calculated during those 999 simulations. The maximum cluster size was set to 10% of the population at risk.

## 3. Results

### 3.1. Demographic Characteristics of Cases

A total of 400 human H7N9 cases were included in our study. Males accounted for 69% of the sample. Ages ranged from less than one year to 91 years, with a median of 58 years. Of the 400 cases, 154 developed symptoms in 2013 and the remainder in 2014. By March 2014, 145 people in the sample had died from the infection, representing a 36.3% mortality rate. Of these deaths, 103 were male and 42 female. They had an age range of 20 to 91 years with a median of 64 years. 65.5% (95/145) of deaths were among people aged 60 years or older.

### 3.2. Temporal Characteristics of Cases

**Figure 1:**
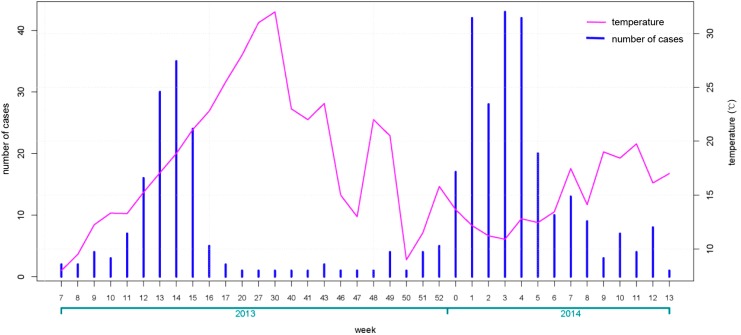
Weekly case number of human infection with avian influenza A (H7N9) virus and average weekly temperature.

As shown in Figure 1, we calculated the number of cases each week with the first cases of H7N9 infection appearing in the eighth week of 2013. There was an initial peak of infection of 37 cases in the 15th week of 2013 followed by a sharp decrease to six or fewer cases per week starting in the 17th week and for the remainder of 2013. A second peak was then observed during a new outbreak in 2014, capping out at 44 cases per week by the end of January. After this, the average number of new cases dropped sharply, averaging little more than five cases per week over the sample period.

### 3.3. Spatial Characteristics of Cases

**Figure 2:**
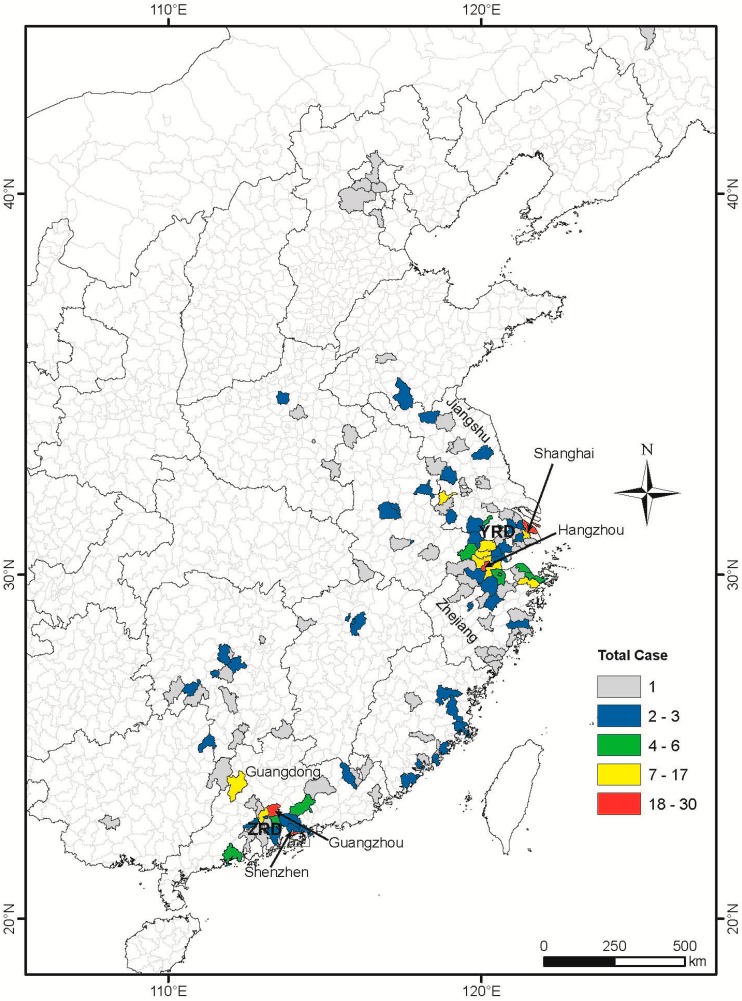
Distribution of H7N9 cases at county level in China from the 19 February 2013 to 31 March 2014. County and province boundary data was accessed from Chinese National Fundamental Geographic Information System (www.nfgis.nsdi.gov.cn) in May 2014. Provinces (Guangdong, Zhejiang and Jiangsu) and cities (Shenzhen, Guangzhou, Hangzhou and Shanghai) mentioned in the paper are marked out. YRD: Yangtze River delta, PRD: Pearl River delta.

Cases of H7N9 infection in the sample covered 14 provinces and 182 counties in China. A distribution map of these cases is shown in Figure 2 with significant spatial clustering in the east and southeast regions of the study area. Most cases occurred in the areas in Shanghai, Jiangsu and Zhejiang provinces near the Yangtze River delta, where infections were first reported, while the remaining cases were centered near the Pearl River delta (Figure 2).

**Figure 3:**
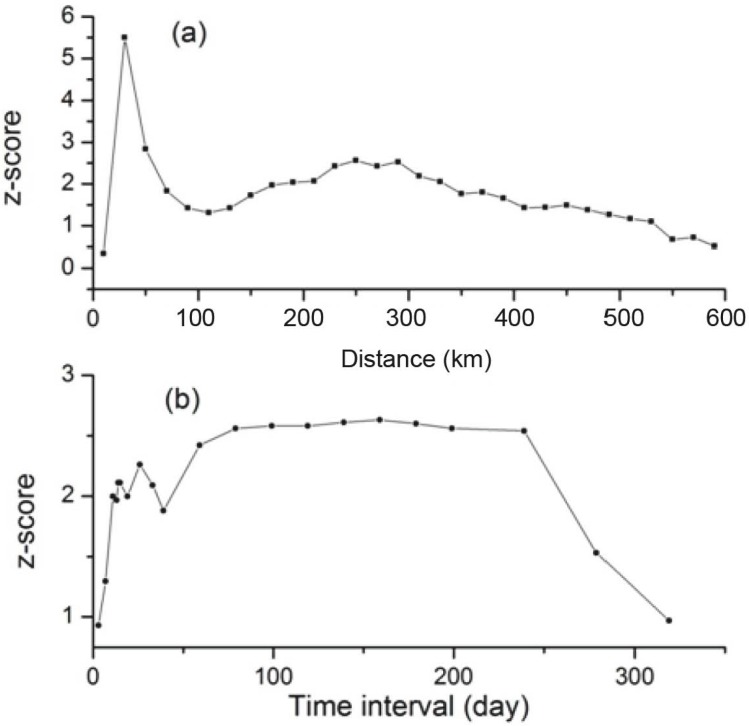
Results of Incremental Spatial Autocorrelation analysis (**a**) and Spatial Autocorrelation analysis (**b**).

A *z*-score peak in the Incremental Spatial Autocorrelation analysis demonstrates a pronounced spatial clustering at distances of 30 and 250 km (Figure 3a). As 30 km is smaller than the distance between the central points of most adjacent counties, using this distance would result in most features being isolated from other features during further analysis. Thus, 250 km was selected as the threshold spatial distance. 

### 3.4. Spatial-Temporal Analysis

**Figure 4:**
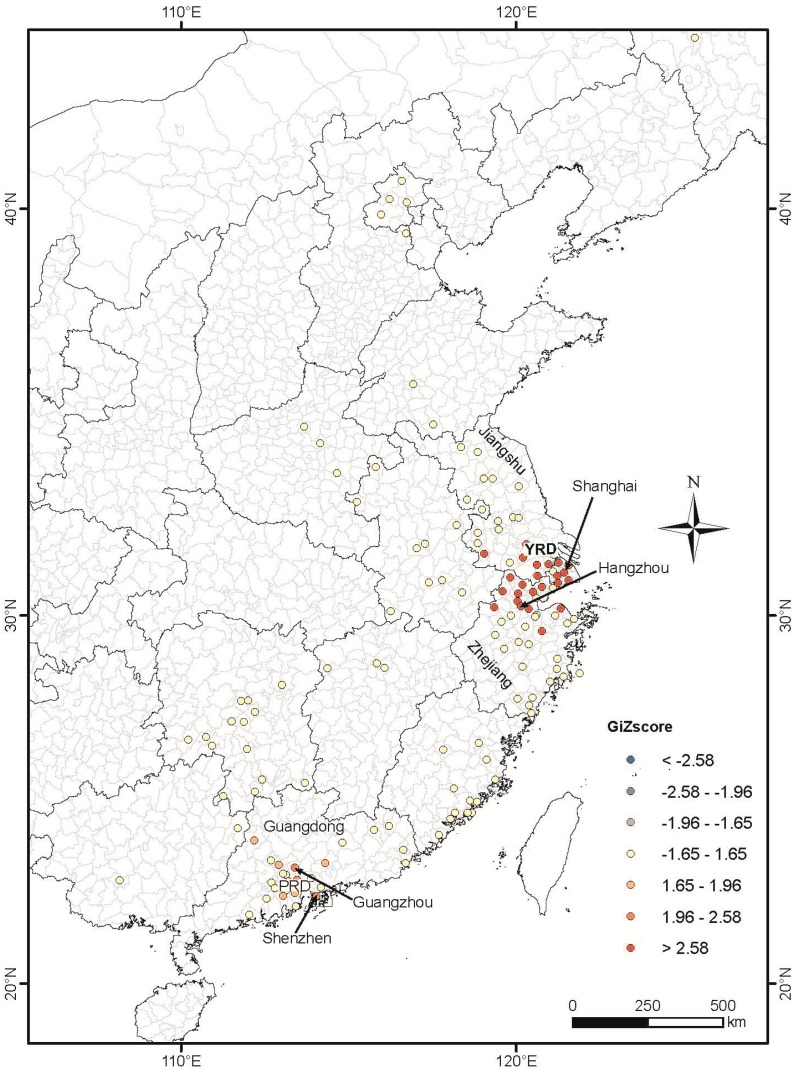
Spatial-temporal clustering of H7N9 cases analyzed using the Hot Spot analysis tool of software ArcMap™ 10.1. See caption in Figure 3 for statistical interpretation of the GiZscore. GiZscore is a measure of the statistical significance of spatial clustering and dispersing. A GiZscore <−2.58 indicates dispersion at *p* = 0.01, −2.58 to −1.96 dispersion at *p* = 0.05, −1.96 to −1.66 dispersion at *p* = 0.1, −1.66 to 1.66 random distribution, 1.66 to 1.96 clustering at *p* = 0.1, 1.96 to 2.58 clustering at *p* = 0.05, and >2.58 clustering at *p* = 0.01.

Spatial Autocorrelation analysis produced a *z*-score peak between 14 and 26 days temporal distance (Figure 3b). Therefore, the temporal distance at which the z-score peak starts, 14 days, was selected as the threshold temporal distance for the space-time Hot-Spot analysis. The result of the analysis is shown in Figure 4.

The result of GIs illustrated statistically significant H7N9 space-time clustering occurred between 26 March and 18 April 2013 near Shanghai and Zhejiang (*p* = 0.01), and between 3 and 4 February 2014 near Guangzhou and Shenzhen (*p* = 0.05). 

**Figure 5:**
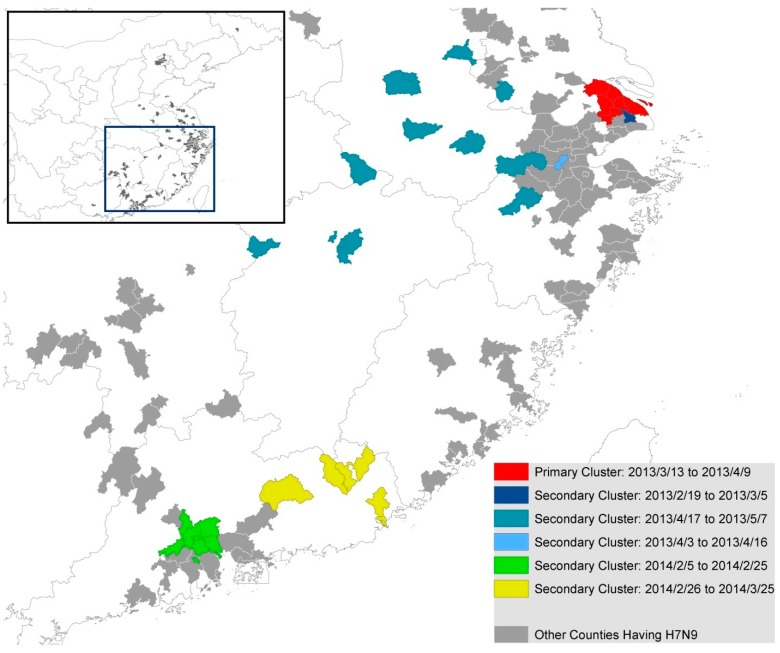
Temporal-spatial clustering of human H7N9 cases detected by SatScan.

The SatScan results also showed that there were six clusters during the whole period. The primary cluster occurred during 13 March 2013 to 9 April 2013, while three secondary clusters occurred during the period of 19 February to 5 March 2013, 3 April to 16 April 2013 and 17 April to 7 May 2013. The remaining two secondary clusters were detected in the year of 2014, from early February to late March (Figure 5). Most of the secondary clusters except those that occurred in Guangzhou and Shenzhen (Figure 2) were identified in counties with no more than three cases during the whole study period. 

Comparing the results of Space-Time scan and ArcGIS, though they are different in some details, good agreement was found in three overall features: (1) the strongest cluster occurred in March 2013 to April 2013 in Shanghai and Zhejiang province; (2) these two areas have no other significant cluster since then during the entire study period; (3) a relatively weak cluster occurs in February 2014 near Guangzhou and Shenzhen.

## 4. Discussion

### 4.1. The Spatial Distribution Characteristics of Human H7N9 Cases

In this study, we used GIS-based space-time analysis to map H7N9 infection cases in China. Our map shows that most human cases occurred in and surrounding the Yangtze River delta region covering Shanghai, Zhejiang and Jiangsu provinces, and in Guangdong province in the Pearl River delta, while sporadic cases were distributed in other provinces. Other studies have illustrated the same cluster pattern, suggesting that the Yangtze River delta could be central to the onset of previous outbreaks [19,20]. Some possible explanations for this include the fact that the Yangtze River and the Pearl River deltas are populated by migrant birds that provide a natural advantage for the influenza A to undergo mutation and reassortment [3]. Previous studies have also shown a positive correlation between human rates of H7N9 infection and the regional density of poultry markets [20,21,22]. While the Yangtze River delta in particular containing large amounts of ducks earmarked for meat and/or egg production [23], it might be one of the reasons that most cases clustered there. Finally, due to a comprehensive surveillance system and generally good medical care in these areas, we cannot ignore the possibility that the increased incidence of H7N9 human infection in these regions is simply a function of better detection rates. However, there are other cities in China, such as Beijing and Hong Kong, being similar to Shanghai in this aspect, but only few cases were detected in these two areas, so this hypothesis is unlikely to be true. 

### 4.2. The Temporal Distribution Characteristics of Human H7N9 Cases

Our results showed that human H7N9 cases are more likely to occur in cool months. Previous study also indicated that high risk was found when the minimum temperature on day 13 prior to disease onset range from 5 to 9 °C and maximum temperature range from 13 to 18 °C [24].

### 4.3. The Spatial-Temporal Distribution Characteristics of Human H7N9 Cases

Only one significant (*p* = 0.01) space-time cluster was identified in the areas on and near the Yangtze River delta (Shanghai, Jiangsu, Zhejiang) between March 2013 and April 2013 during the entire study period. A similar cluster is identified if the H7N9 case dataset was analyzed for space-time clustering using SatScan (see SatScan result; Figure 5). It is interesting to note that most cases in 2014 do not cluster when time is taken into account. One explanation for this is that the central government intervention to manage H7N9 encouraged local officials and government agencies to shut down poultry markets where H7N9 cases were reported in nearby areas [25,26]. For example, during the outbreak in April 2013, government intervention resulted in the shutting down of 780 live poultry markets [27]. Though sporadic H7N9 cases still occurred after May 2013 with a large resurgence in 2014, space-time clustering is less significant (*p* = 0.05) (Figure 5) and spans over smaller area and shorter period than clustering in 2013. This suggests that shutting down poultry markets can be an effective intervention in halting the spread of H7N9. 

In fact the effectiveness of this intervention has been demonstrated in a previous study where it was shown that the closure of live poultry markets reduced the mean daily number of human infections of H7N9 cases by approximately 97% in four major cities [28].

### 4.4. Study Limitations

H7N9 almost always causes severe disease rather than subclinical infections although there have been reports of mild or asymptomatic cases [28,29,30]. This variation in case presentation may have led to a potential bias in screening and surveillance despite China having had an effective surveillance and reporting system since 2003. 

## 5. Conclusions

Our results demonstrate that there is the strongest cluster occurred in March 2013 to April 2013 in Shanghai and Zhejiang provinces and these two areas have no significant cluster since then during the entire study period. Besides, a relatively weak cluster occurs in February 2014 near Guangzhou and Shenzhen. This suggests that disease control polices probably have effectively prevented the spreading of H7N9 virus.
